# Optical and structural properties of amorphous Se_*x*_Te_100-*x*_ aligned nanorods

**DOI:** 10.1186/1556-276X-8-520

**Published:** 2013-12-09

**Authors:** Faisal A Al-Agel

**Affiliations:** 1 Department of Physics, King Abdulaziz University, Jeddah 21589, Saudi Arabia

**Keywords:** Chalcogenides, a-Se_*x*_Te_100-*x*_, Melt quenching, Vacuum evaporation, Thin films, dc conductivity, Activation energy, Absorption coefficient, Optical band gap, Optical constants

## Abstract

In the present work, we report studies on optical and structural phenomenon in as-deposited thin films composed of aligned nanorods of amorphous Se_*x*_Te_100-*x*_ (*x* = 3, 6, 9, and 12). In structural studies, field emission scanning electron microscopic (FESEM) images suggest that these thin films contain high yield of aligned nanorods. These nanorods show a completely amorphous nature, which is verified by X-ray diffraction patterns of these thin films. Optical studies include the measurement of spectral dependence of absorption, reflection, and transmission of these thin films, respectively. On the basis of optical absorption data, a direct optical band gap is observed. This observation of a direct optical band gap in these nanorods is interesting as chalcogenides normally show an indirect band gap, and due to this reason, these materials could not become very popular for semiconducting devices. Therefore, this is an important report and will open up new directions for the application of these materials in semiconducting devices. The value of this optical band gap is found to decrease with the increase in selenium (Se) concentration. The reflection and absorption data are employed to estimate the values of optical constants (extinction coefficient (*k*) and refractive index (*n*)). From the spectral dependence of these optical constants, it is found that the values of refractive index (*n*) increase, whereas the values of extinction coefficient (*k*) decrease with the increase in photon energy. The real and imaginary parts of dielectric constants calculated with the values of extinction coefficient (*k*) and refractive index (*n*), are found to vary with photon energy and dopant concentration.

## Background

Amorphous semiconductors have been known for years, and a lot of work on the applications of these materials is available in the literature [1,2]. Among these materials, chalcogenides are the most studied materials. In fact, amorphous materials became popular only after the discovery of chalcogenides, and later, many interesting physical properties of these materials [3,4] were reported. These chalcogenides have special application in optical devices due to their transparency in the IR region. They are also used in switching and memory devices, and the most popular application of these materials is in phase change recording [5,6]. Among the chalcogen family, selenium and tellurium have been studied widely due their potential applications [7,8]. Glassy selenium is one of the popular materials for the development of various solid-state devices such as electrophotographic and switching and memory devices [9]. For the last few years, tellurium-rich alloys attracted a lot of attention due to their potential applications in data storage devices [10,11]. It is well understood that the bonding between Se and Te is weaker than the Se-Se bonds due to the catalytic effect of tellurium on the crystallization of selenium. Several workers [12-14] reported that tellurium-rich glasses have good transparency in the infrared and high refractive index, which makes these glasses important for optical devices also.

Tellurium-rich glassy alloys of Se-Te are widely used for commercial, scientific, and technological purposes. Their application ranges from optical recording media to xerography [15-17]. Khan et al. [18] studied the electrical and optical properties of thin films of a-Se_*x*_Te_100-*x*_ system. They reported an indirect optical band gap and electrical transport via a thermally activated process in this system. Salah et al. [19] studied the thin films of polycrystalline Te_94_Se_6_ nanoparticles. Further, they prepared these nanoparticles at different working gas pressures and studied the pressure dependence of optical band gap in these nanoparticles. They reported that a direct optical band gap and the values of optical band gap are found to be pressure dependent. Salah et al. [20] deposited thin films composed of nanoparticles of polycrystalline Se_*x*_Te_100-*x*_ and studied the optical properties of these nanoparticles. They reported a direct optical band gap in this system, and the values of optical band gap are found to be size and composition dependent. In the present work, we have also studied a-Se_*x*_Te_100-*x*_ system and produced aligned nanorods of this alloy. The optical and structural properties of these well-aligned nanorods are studied. In our case, we found that these nanorods are aligned and their structure is completely amorphous. These amorphous nanorods show an enhanced and direct band gap as compared to the reported results on polycrystalline materials [19,20]. These findings in the field of nanochalcogenide glasses will be interesting for applications in devices as these materials are cost-effective, and fabricating devices using these materials will also reduce the cost of devices. It is also important to understand the optical phenomenon in a-Se_*x*_Te_100-*x*_ nanorods as reduction in the size of the material (nanoscale) may result in a dramatic change in the properties. Keeping the above facts in view, it is therefore extremely important to study the properties of as-prepared a-Se_*x*_Te_100-*x*_ aligned nanorods.

## Methods

Thin films of a-Se_*x*_Te_100-*x*_ were deposited using a rapid thermal evaporation technique. In this method, as-prepared alloys were evaporated in an argon gas environment. Thermal evaporation was modified to rapid thermal evaporation by constructing a small sub-evaporation chamber using a quartz tube that is 30 mm in diameter and 110 mm in length. An arrangement was made in this quartz tube for the gas inlet, opening of the evaporation source, sample holder, and the gas outlet. With the quartz tube, we were able to confine the evaporated material and maintain a uniform gas pressure in the vicinity of the evaporation source. A molybdenum boat was used as an evaporation source. For depositing the thin films, the glass substrate was pasted at the top of the tube. Film thickness was measured with a quartz crystal thickness monitor (FTM 7, BOC Edwards, West Sussex, UK). After loading the glass substrate and the source material, the chamber was evacuated to 10^-5^ Torr. The inert gas (Ar) with 0.1 Torr pressure was injected into the sub-chamber, and the same gas pressure was maintained throughout the evaporation process. Once a thickness of 500 Å was attained, the evaporation source was covered with a shutter, which was operated from outside. After the process was over, thin films were taken out of the chamber and were analyzed for structural and optical properties. X-ray diffraction patterns of thin films of a-Se_*x*_Te_100-*x*_ nanorods were obtained with the help of an Ultima-IV (Rigaku, Tokyo, Japan) diffractometer (*λ* = 1.5418 Å wavelength CuKα radiation at 40 kV accelerating voltage and 30 mA current), using parallel beam geometry with a multipurpose thin film attachment. X-ray diffraction (XRD) patterns for all the studied thin films were recorded in theta - 2 theta scans with a grazing incidence angle of 1°, an angular interval (20° to 80°), a step size of 0.05°, and a count time of 2 s per step. Field emission scanning electron microscopic (FESEM) images of these thin films containing aligned nanorods were obtained using a Quanta FEI SEM (FEI Co., Hillsboro, OR, USA) operated at 30 kV. A 120-kVtransmission electron microscope (TEM; JEM-1400, JEOL, Tokyo, Japan) was employed to study the microstructure of these aligned nanorods. Energy-dispersive spectroscopy (EDS) was employed to study the composition of these as-deposited films using EDAX (Ametek, Berwyn, PA, USA) operated at an accelerating voltage of 15 kV for 120 s.

To study the optical properties of these samples, we deposited the a-Se_*x*_Te_100-*x*_thin films on the glass substrates at room temperature using a modified thermal evaporation system. The thickness of the films was kept fixed at 500 Å, which was measured using the quartz crystal thickness monitor (FTM 7, BOC Edwards). The experimental data on optical absorption, reflection, and transmission was recorded using a computer-controlled JascoV-500UV/Vis/NIR spectrophotometer (Jasco Analytical Instruments, Easton, MD, USA). It is well known that we normally measure optical density with the instrument and divide this optical density by the thickness of the film to get the value of the absorption coefficient. To neutralize the absorbance of glass, we used the glass substrate as a reference as our thin films were deposited on the glass substrate. The optical absorption, reflection, and transmission were recorded as a function of incident photon energy for a wavelength range (400 to 900 nm).

## Results and discussion

**Figure 1:**
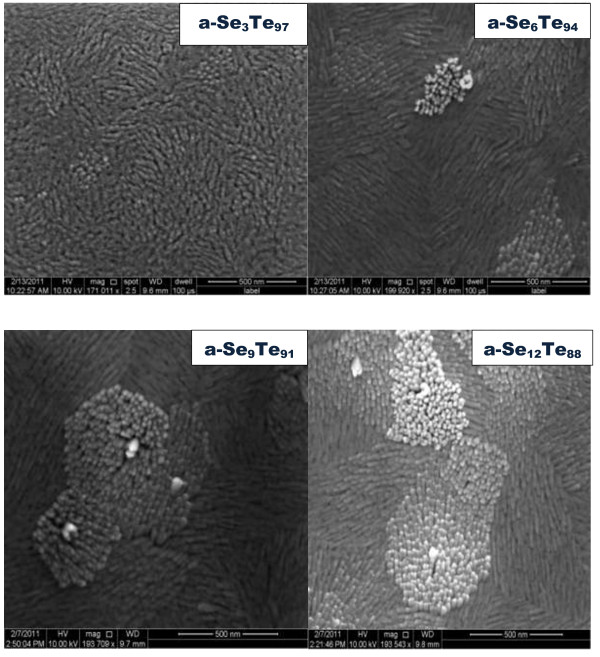
**FESEM images of thin films composed of a-Se**_***x***_**Te**_**100**-***x ***_**aligned nanorods.**

**Figure 2:**
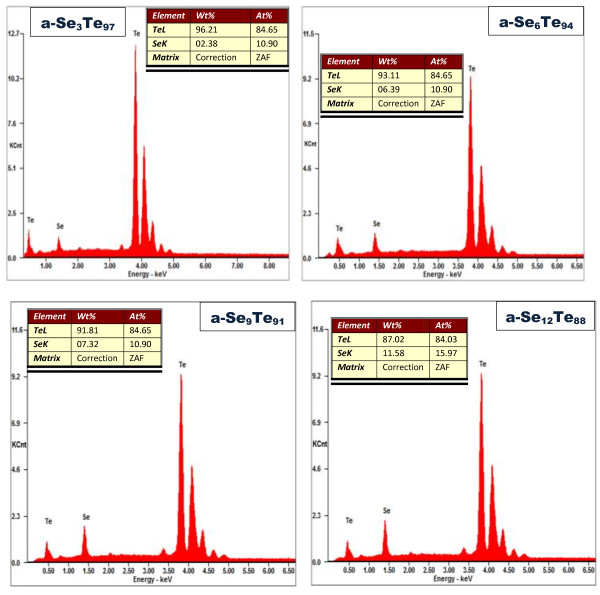
**EDS spectra of a-Se**_***x***_**Te**_**100**-***x ***_**thin films.**

**Figure 3:**
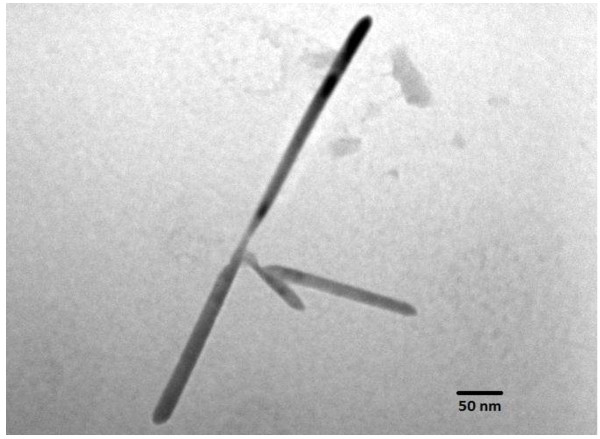
**TEM image of a-Se**_**9**_**Te**_**91**_**nanorod.**

**Figure 4:**
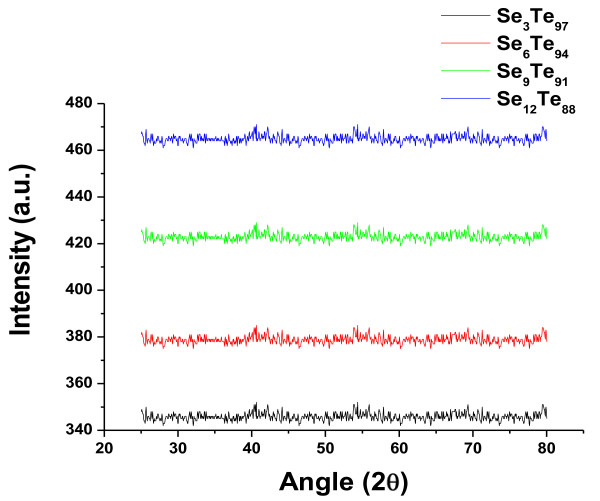
**XRD pattern of a-Se**_***x***_**Te**_**100**-***x***_**.**

The morphology of the a-Se_*x*_Te_100-*x*_ thin films is studied using FESEM. Figure 1 shows FESEM images of a-Se_*x*_Te_100-*x*_ thin films. It is evident from these images that Se_*x*_Te_100-*x*_ thin films contain high yield of aligned nanorods. These nanorods are very short but perfectly aligned. The diameter of these nanorods is between 10 and 20 nm, and the length is in the order of several hundred nanometers. We have included the FESEM images for all the studied compositions of a-Se_*x*_Te_100-*x*_ thin films. It is evident from these images that the nucleation of nanorods starts in the first sample, i.e., a-Se_3_Te_97_, and an increase in the concentration of Se results in the growth of nanorods. The yield of the nanorods increases with the increase in selenium concentration. The composition of these as-prepared alloys has also been verified using EDS. It is observed that the set composition of the alloys is very close to the composition of as-prepared alloys. The EDS spectra for the a-Se_*x*_Te_100-*x*_ thin films are presented in Figure 2. This shows the close agreement with the final composition and set composition of this alloy. The microstructure of these aligned nanorods is studied by a TEM operated at 100 kV, and the TEM image of a single nanorod is presented in Figure 3. From this image, it is clear that the length of the nanorod is of the order of several hundred nanometers, and the diameter is approximately 20 nm. Figure 4 presents the XRD patterns of a-Se_*x*_Te_100-*x*_ alloys. From the XRD patterns, we have not seen any significant peak for the present sample of nanorods. It is therefore concluded that these samples are amorphous in nature. The growth mechanism of these nanorods can be explained by the inert gas condensation method. In this method, a small quantity of as-prepared glassy alloy in powder form is kept in a molybdenum boat, and then, a vacuum of the order of 10^-6^ Torr is maintained in the chamber as well as in the quartz tube. Finally, an inert gas (argon) is purged into the tube. The flow of the gas is maintained in such a way that the pressure inside the quartz tube remains at 0.1 Torr throughout the process. Under these controlled conditions, the glassy alloys are evaporated in the presence of ambient argon gas atmosphere in the chamber to obtain the aligned nanorod deposit in thin film form. Here, argon is used as an inert gas in the tube, and its role is to offer frequent collisions to the atoms of the evaporated materials. These frequent collisions of atoms result in the reduction of energy of the evaporated atoms. In this process, the material is typically vaporized into a low-density gas (inert gas), and the vapors move from the hot source to the glass substrate, which is pasted at the top of the tube. The substrate is kept at a much lower temperature as compared to evaporation temperature. Due to this temperature difference, the deposition efficiency will be enhanced. The evaporated material is deposited on a glass substrate pasted at the top of the quartz tube. It is worth mentioning here that since the film of glassy alloy is deposited at a low substrate temperature, the material is further quenched. This makes the present sample highly amorphous.

On the basis of experimentally recorded data, we calculated the values of absorption coefficient (*α*). To calculate these values, we employ the following equation:

(1) a=OD/t

where OD is the optical density measured for a given film thickness (*t*).

From the spectral dependence of absorption coefficient (*α*), we found an increase in the value of absorption coefficient (*α*) with the increase in photon energy for the a-Se_*x*_Te_100-*x*_ thin films. For this system of aligned nanorods, the calculated values of the absorption coefficient are of the order of ~10^5^ cm^-1^. This is comparable with the reports of other workers presented in the literature [18-21].

To understand the absorption process in amorphous semiconductors, there are three popular processes, namely residual below-gap absorption, Urbach tails, and inter-band absorption. The absorption observed in the amorphous materials can be explained with the help of any of these processes. It is well known that amorphous materials especially chalcogenides show highly reproducible optical edges. These edges are found to be relatively insensitive to preparation conditions. The observable absorption with a gap under equilibrium condition fits well only with the first process for such type of materials [22]. In other glassy materials, a different type of optical absorption edge is observed. In these materials, we normally observe an exponential increase in the value of the absorption coefficient with the increase in photon energy near the gap [23]. In our case, we have observed a similar behavior, and the typical absorption edge is represented as the Urbach edge, which is presented by the following relation:

(2) $$a\;\sim \;\exp\;\left[A\left(\mathrm{h\nu}\;-\;h\nu_{0}\right)\right]\;/\;\mathrm{KT}$$

where *A* is a constant of the order of unity and ν_0_ is the constant corresponding to the lowest excitonic frequency.

Mostly, the fundamental absorption edge observed in amorphous semiconductors follows an exponential law. In such cases, the absorption coefficient obeys the following relation:

(3) $${\left(a.\mathrm{h\nu}\right)}^{1/n}\;=\;B\;\left(\mathrm{h\nu}-\;E_{g}\right)$$

where ν is the frequency of the incident beam (*ω* = 2*π*ν), *B* is a constant, *E*_g_ is the optical band gap, and *n* is an exponent. This exponent can have different values, i.e., 1/2, 3/2, 2, or 3, depending on the nature of electronic transition responsible for the absorption. For allowed direct transition, we take *n* as 1/2 for allowed direct transition and as 3/2 for forbidden direct transition, whereas for allowed indirect transition, *n* is taken as 2. In our case, we observed the allowed direct transition, and we take *n* to be equal to 1/2 [24,25].

To determine the nature of electronic transition for a-Se_*x*_Te_100-*x*_, we tried to fit the experimental data with different values of *n*, and it is found that the data gives the best fit with the value of *n* to be equal to 1/2. This suggests that this sample of nanorods shows direct electronic transition, and this direct transition can be expressed in terms of optical gap, optical absorption coefficient (*α*), and the energy (hν) of the incident photon, which is presented as

(4) $${\left(a.\mathrm{h\nu}\right)}^{2}\propto \left(\mathrm{h\nu}\;-\;E_{g}\right).$$

**Figure 5:**
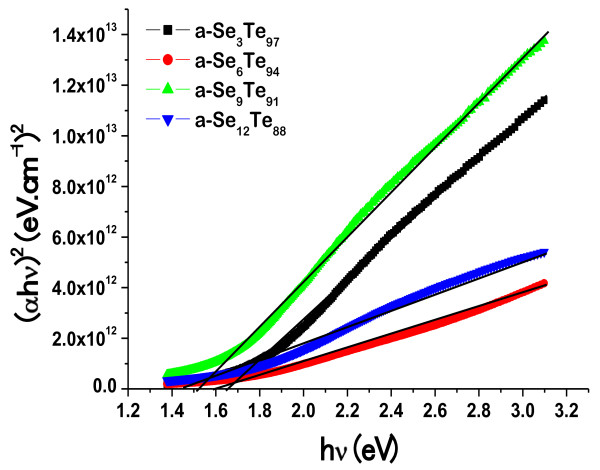
**(*α*** **hν)**^**2 **^**against photon energy (hν) in a-Se**_***x***_**Te**_**100-** ***x ***_**thin films composed of aligned nanorods.**

Using the above relation, we plot (*α*.hν)^2^ vs. photon energy (hν) for the present case, and the experimental data is fitted with the best fit line. The extrapolation of the line on the *x*-axis gives the value of direct optical band gap (*E*_g_). The plot showing the variation of (α.hν)^2^ with photon energy (hν) is presented in Figure 5 for the present system of a-Se_*x*_Te_100-*x*_ films composed of aligned nanorods. The values of *E*_g_ calculated for each sample of a-Se_*x*_Te_100-*x*_ thin films are shown in Table 1. For this system of nanorods, the value of optical band gap (*E*_g_) is found to decrease from 1.66 to 1.45 eV with increasing Se content in a-Se_*x*_Te_100-*x*_ thin films. Khan et al. [18] studied the electrical and optical properties of as-deposited a-Se_*x*_Te_100-*x*_ thin films (*x* = 3, 6, 9, and 12). FESEM images show that the thin films contain clusters of particles. The size of these particles varies between 100 and 300 nm. They observed an indirect optical band gap in this system, which decreases from 1.29 to 1.03 eV on increasing Se concentration from *x* = 3 to *x* = 12. They have also reported a significant change in the value of the optical constants with the change in Se concentration. In our case, we have studied the structural and optical properties of a-Se_*x*_Te_100-*x*_ thin films (*x* = 3, 6, 9, and 12) containing aligned nanorods. Here, thin films have been synthesized by different techniques. FESEM images reveal that these thin films contain high yield of aligned nanorods with diameter in the range of 10 to 30 nm. Therefore, the size is reduced from several hundred nanometers in the previous case to few tens of nanometers in our case. Due to this size reduction, the optical properties show a dramatic change and the optical band gap becomes direct with enhanced value as compared to the observation of an indirect band gap in the previous case. The values of optical constants (refractive index and extinction coefficient) are also enhanced significantly as compared to results from a previous report [18]. The values of optical band gap and optical constants are enhanced and decreased with the increase in selenium concentration. This enhancement in the value of optical band gap and optical constants will be attributed to the phenomena of size effect. Salah et al. [26] studied Se_35_Te_65-*x*_Ge_*x*_ (*x* = 0, 3, 6, 9, and 12) nanoparticle thin films. They reported that the values of indirect optical band gap (*E*_g_) were found to decrease from 0.83 to 0.69 eV by increasing the concentration of Ge from 0 to 12. The values of optical constants also showed significant change with the increase in selenium concentration. Khan et al. [27] studied the electrical and optical properties of a-Se_70_Te_30_nanorod thin film. They reported that the absorption mechanism was due to indirect transition. The optical band gap was estimated to be 1.18 eV. Khan et al. [28] observed an indirect band gap in the tellurium-rich Ga_10_Te_90-*x*_Sb_*x*_ (*x* = 5, 10, 20, and 30) thin films. The value of band gap decreased with an increase in Sb content. Ilyas et al. [29] also reported an indirect band gap in the tellurium-rich Ga_*x*_Te_100-*x*_ thin films. Abd-Elrahman [30] studied the effect of composition on the optical constants of Se_100-*x*_Te_*x*_ (*x* = 30, 50, and 70) chalcogenide thin films. They reported that an increase in Se contents (from *x* = 30 to*x* = 70) resulted in an increase in indirect gap from 1.33 to 1.85 eV. They also found that the absorption coefficient, refractive index, extinction coefficient, and dispersion energy of the films were dependent on the film composition. El-Zahed et al. [31] studied the dependence of optical band gap with the composition of Se_(1-*x*)_Te_*x*_ (*x* = 0.2, 0.4, 0.5, and 0.8). They found that the optical gap was a function of composition and the width of optical gap varied from 1.8 to 1.06 eV. The band gap decreased with increasing Te content. Most of the reports presented above predicted indirect band gap and the compositional and photon energy dependence of optical band gap and optical constants in the chalcogenides, whereas in present work, size reduction to the nanoscale level results in a dramatic change in the optical properties. Therefore, it may be concluded that the results presented in this paper show the effect of size on optical properties, i.e., observation of direct band gap and enhanced value of band gap and optical constants for the a-Se_*x*_Te_100-*x*_thin films containing aligned nanorods.

**Table 1:** **Optical parameters of a-Se**_***x***_**Te**_**100-** ***x***_**thin films at 600 nm**

**Sample**	***E***_**g**_**(eV)**	***α*** **(cm**^**-1**^**)**	***k***	***n***	**ε**_***r***_ ***′***	**ε**_***r***_ ***″***
Se_3_Te_97_	1.66	8.40 × 10^5^	4.01	11.90	125.58	95.53
Se_6_Te_94_	1.59	5.16 × 10^5^	2.47	10.69	88.54	108.72
Se_9_Te_91_	1.51	10.6 × 10^5^	5.08	9.08	66.31	72.85
Se_12_Te_88_	1.45	6.50 × 10^5^	3.11	5.54	20.98	34.40

It is well understood that the optical absorption is dependent on both the short-range order and defect states observed in amorphous systems. We can employ Mott and Davis's ‘density of state model’ to explain this decrease in optical band gap with the increase in Se concentration. It was suggested by Mott and Davis [32] that the degree of disorder and defects in the amorphous systems are two major factors affecting the width of the localized states near the mobility edges. For the present case of a-Se_*x*_Te_100-*x*_ thin films, it is proposed that the unsaturated bonds together with some saturated bonds are produced during the deposition of atoms in the present as-deposited films [33]. It is well known that the as-deposited chalcogenide thin films always contain a high concentration of unsaturated bonds or defects. These defects are responsible for the presence of localized states in the amorphous band gap. Therefore, these unsaturated bonds result in the formation of defects in the presently studied thin films containing aligned nanorods, thereby producing a large number of localized/defect states in the present system. Tellurium glass contains short chains, whereas selenium glass contains long chains and selenium rings. As Se concentration increases or Te concentration decreases, the number of Se rings increases and the number of long Se-Te polymeric chains and Se-Te mixed rings decreases [34]. Therefore, the addition of selenium to tellurium increases the number of defect states, which increases further with the increase in Se concentration. As these defect states are also associated with unsaturated bonds formed during the deposition of these thin films, we may state that the number of unsaturated bonds increases with the increase in Se concentration. This increase in the defect states or unsaturated bonds with the concentration of Se results in the narrowing of optical band gap. Therefore, the optical band gap in the present system decreases with the increase in Se concentration. We can also interpret this decrease in optical band gap with respect to the shift in Fermi level. The position of Fermi level in such systems is determined by the distribution of electrons over the localized states [35].

For the present system of a-Se_*x*_Te_100-*x*_ thin films containing aligned nanorods, we use the following relation to estimate the values of extinction coefficient (*k*). This relation is given as

(5) a=4πk/λ.

We use the theory of reflectivity of light to estimate the values of refractive index (*n*) and extinction coefficient (*k*) for the present system. Employing this theory, the reflectance of light from a thin film can be written in terms of Fresnel's coefficient. Therefore, the reflectivity on an interface can be expressed by the following relation [36-38]:

(6) $$R\;=\;\left[{\left(n-1\right)}^{2}\;+\;k^{2}\right]/\left[{\left(n+1\right)}^{2}\;+\;k^{2}\right],$$

Where *λ* is the wavelength of the incident light and *α* is the absorption coefficient.

**Figure 6:**
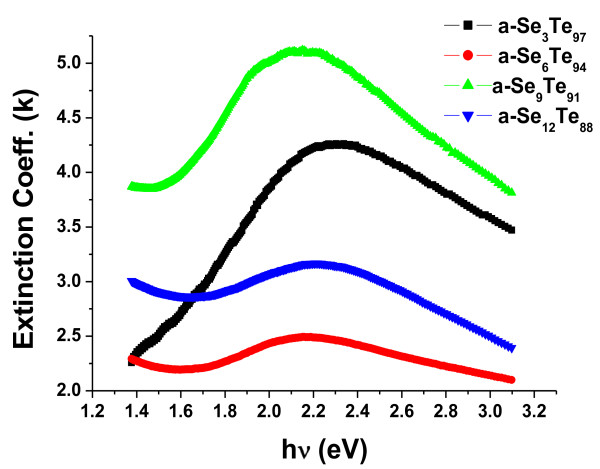
**Variation of extinction coefficient (*k*) with incident photon energy (hν) in a-Se**_***x***_**Te**_**100-** ***x ***_**thin films composed of aligned nanorods.**

**Figure 7:**
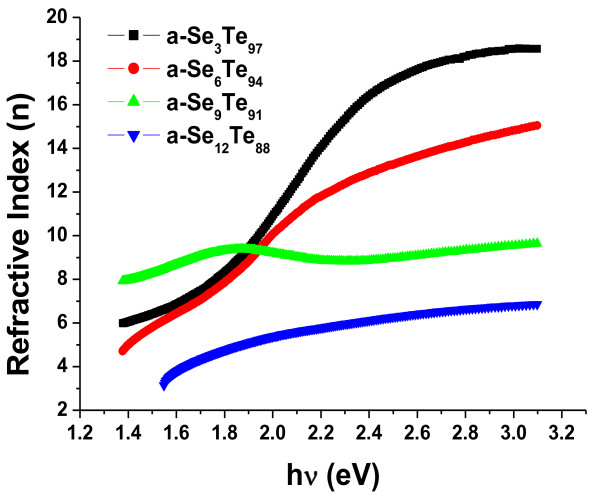
**Variation of refractive index (*n*) with incident photon energy (hν) in a-Se**_***x***_**Te**_**100-** ***x ***_**thin films composed of aligned nanorods.**

The dependence of incident photonic energy on the extinction coefficient (*k*) for Se_*x*_Te_100-*x*_ thin films containing aligned nanorods is shown in Figure 6. It is observed that the value of extinction coefficient shows an overall decreasing trend with the increase in photon energy. Figure 7 presents the variation of refractive index (*n*) with the photon energy. From this figure, an increase in the value of refractive index with the increase in photon energy is observed. These results are in close agreement with the results reported by various workers [18,39]. The calculated values of *n* and *k* for different compositions of Se are shown in Table 1. From this table, it is observed that the values of refractive index (*n*) and extinction coefficient (*k*) decreases with the increase in Se content for Se_*x*_Te_100-*x*_ thin films containing aligned nanorods. This spectral and dopant dependence of optical band gap and optical constants with the photon energy will be helpful in deciding on the suitability of this system of aligned nanorods for application in optical data storage devices.

**Figure 8:**
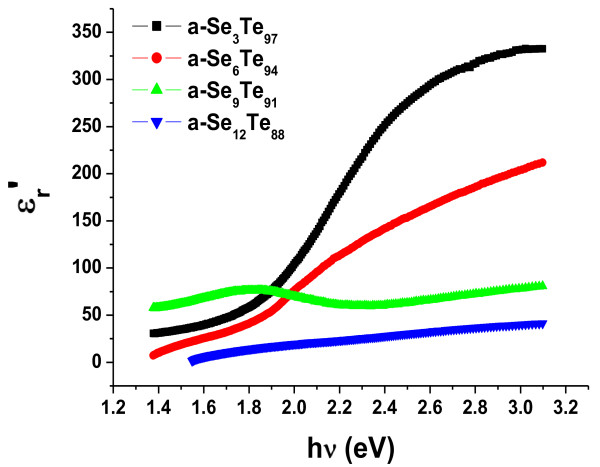
**Variation of dielectric constant real part with incident photon energy in a-Se**_***x***_**Te**_**100-*x***_**aligned nanorod thin films. ***Є*_*r*_′, real part of the dielectric constant; hν, incident photon energy.

**Figure 9:**
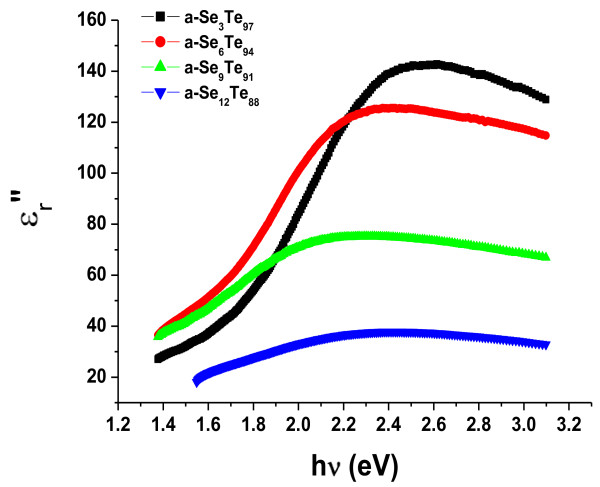
**Variation of dielectric constant imaginary part with incident photon energy in a-Se**_***x***_**Te**_**100-*x***_**aligned nanorod thin films.** Є_*r*_″, imaginary part of the dielectric constant; hν, incident photon energy.

Using the values of refractive index (*n*) and extinction coefficient (*k*) obtained using the above mentioned relations, we have calculated the values of the real part (*Є*_*r*_′ = *n*^2^ – *k*^2^) and imaginary part (*Є*_*r*_″ = 2*nk*) of the dielectric constant, and their variation with photon energy is presented in Figures 8 and 9. The calculated values of the real part and imaginary part of the dielectric constant are also presented in Table 1. These are found to increase with the increase in photon energy, whereas the values of these parameters are observed to decrease on the addition of Se impurity in the present system of Se_*x*_Te_100-*x*_ thin films.

In the case of compound semiconductors deposited from the vapor, we may consider the possibility of like bonds. In III-V compounds, we may consider two types of like bonds, which are taken as two possible anti-site defects. In such cases, chemical disorder produces large change in potential through the Coulombian interaction due to large ionic contribution to the bonding. Theye [33] reported that the bonding in glassy materials is covalent and the chemical disorder results only in small changes in the local potential.

These direct band gap materials may have potential applications in optical recording media, xerography, electrographic applications, infrared spectroscopy, and laser fibers. Moreover, their transparency in the infrared region and their high refractive index are good indicators for integrated optics and detection in the mid- and thermal infrared spectral domain. The observance of a direct band gap in this material is very interesting and will open up new direction for applications in nanodevices. Since the popular direct band gap materials, e.g., GaAs, GaN, InAs, and InP, are more expensive as compared to chalcogenides and most of the industries are facing problems in reducing the cost of the devices due to the high cost of these materials, the chalcogenides being a cheap material will provide a good option for industries to produce cost-effective devices.

## Conclusions

The amorphous nature of Se_*x*_Te_100-*x*_ thin films (*x* = 3, 6, 9, and 12) is predicted with the help of XRD patterns. On the basis of the best fitting of optical absorption data, it is suggested that the band gap follows direct optical transitions and its value decreases on adding the Se content to the presently studied system. One of the possible reasons behind this decrease in band gap may be due to the increase in the disorderedness of the system, which results in an increase in the density of defect states. The value of refractive index increases with the increase in photon energy, whereas the value of extinction coefficient decreases with the increase in photon energy and Se concentration. The calculated values of real and imaginary parts of dielectric constants are found to decrease with the increase in Se content for the present system. On the basis of the above reported values of optical parameters, one may decide the suitability of these nanorods for optical devices.

## Competing interests

The author declares no competing interests.
